# The In Vitro Multifaceted Biological Activity of Catechins in Relation to Their Oxidation Potentials

**DOI:** 10.3390/molecules31081328

**Published:** 2026-04-17

**Authors:** Małgorzata Wronkowska, Danuta Zielińska, Małgorzata Starowicz, Mateusz Szydłowski, Mariusz Konrad Piskuła, Henryk Zieliński

**Affiliations:** 1 InLife Institute of Animal Reproduction and Food Research, Polish Academy of Sciences, Trylińskiego 18, 10-683 Olsztyn, Poland; m.wronkowska@pan.olsztyn.pl (M.W.); m.starowicz@pan.olsztyn.pl (M.S.); m.szydlowski@pan.olsztyn.pl (M.S.); m.piskula@pan.olsztyn.pl (M.K.P.); 2 Department of Chemistry, University of Warmia and Mazury in Olsztyn, Plac Łódzki 4, 10-721 Olsztyn, Poland

**Keywords:** catechins, antioxidant potency, antiglycation activity, angiotensin-converting enzyme inhibition, acetylcholinesterase inhibition

## Abstract

In this study, the rank of multifaceted activity of catechin (**C**), epicatechin (**EC**), epigallocatechin (**EGC**), epicatechin-3-gallate (**ECG**) and epigallocatechin-3-gallate (**EGCG**) was addressed. Their antioxidant activity was determined by the differential pulse voltammetry (DPV), whereas their ability to inhibit angiotensin-converting enzyme (ACE) activity, acetylcholinesterase activity (AChE), and formation of the advanced glycation end-products (AGEs) was performed in a model system to show their importance against hypertension, Alzheimer-type dementia, and diabetic’s complication, respectively. The order of the antioxidant potential of catechins in comparision to gallic acid (GA) was **EGCG** > **ECG** > **EC** > **EGC** ≈ **C** > **GA**, whereas the order of the ACE inhibitory activity was **EGCG** > **ECG** > **EGC** > **EC** > **C**, thus indicating the importance of the structure–activity relationship. The correlation between IC_50_ for ACE inhibition of catechins and their antioxidant activity had the value r = −0.60. The order of the AChE enzyme inhibitory activity was **EGCG** ≈ **EGC** > **ECG** > **EC** > **C**, and the weak positive correlation between IC_50_ and the first anodic peak potential (E_pa1_) values was noted (r = 0.67). The ranking of the anti-AGE activities was **EGCG** ≈ **ECG** > **EGC** > **EC** > **C**, and a negative correlation between the inhibitory activity of catechins against AGE formation and their antioxidant activity was r = −0.82, whereas a positive correlation (r = 0.88) was noted between their first anodic peak potential (E_pa1_) values. The provided results expand our knowledge on the multifaceted activity of catechins, indicating **EGCG** and **ECG** as the most active antioxidants against inhibition of ACE and AChE as well as towards AGE formation.

## 1. Introduction

The group of catechins (flavan-3-ol) belonging to the group of flavonoids include (+)-catechin (**C**), (-)-epicatechin (**EC**), (-)-epigallocatechin (**EGC**), (-)-epicatechin-3-gallate (**ECG**), and (-)-epigallocatechin-3-gallate (**EGCG**). The best source of catechins is unfermented green tea [1]. Green tea has been considered a medicine and a healthful beverage since ancient times. Traditional Chinese medicine has recommended this plant for headaches, body aches and pains, digestion, depression, detoxification, as an energizer and, in general, to prolong life. Catechins also occur naturally in black tea, coffee, berries, grapes, and wine. Catechins are in vitro and in vivo strong antioxidants responsible for the scavenging of reactive oxygen species, inhibition of the formation of free radicals, and lipid peroxidation. Available literature data indicate that the antioxidant activity of catechins contained in green tea and their significant impact on the prevention of civilization diseases are largely dependent on the presence of structural groups in the molecules, as well as the number of hydroxyl groups [2]. In this context, the green tea catechins are interesting compounds due to their widespread popularity all over the world [3]. Anti-inflammatory and antioxidant properties, as well as chemo-preventive activity and prevention effects, in various types of cancers are considered the most important action of the catechin group [3,4,5,6,7,8,9]. Recently Kuban-Jankowska et al. [10] showed that epigallocatechin and epigallocatechin gallate were the most effective inhibitors of MCF-7 breast cancer cell viability. They also observed that epigallocatechin, epigallocatechin gallate, epicatechin, and epicatechin gallate may decrease enzymatic activity of PTP1B phosphatase. PTP1B is a tyrosine phosphatase that is oxidative stress-regulated and is involved with prooncogenic pathways, leading to the formation of a.o. breast cancer. These evidence support the previous observation on the activity of catechins, mainly epigallocatechin and epigallocatechin gallate, against breast cancer progression.

**Figure 1:**
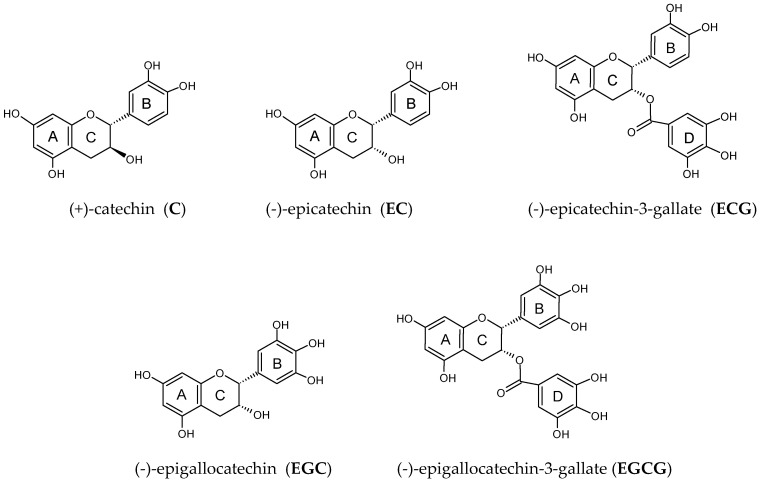
Structures of the investigated compounds: (+)–catechin (**C**), (-)-epicatechin (**EC**), (-)-epigallocatechin (**EGC**), (-)-epicatechin-3-gallate (**ECG**), and (-)-epigallocatechin-3-gallate (**EGCG**).

Available data also indicate the antioxidant [5], anti-inflammatory [11], anti-microbial, anti-viral, anti-diabetic, anti-obesity, and hypotensive effects of catechins [12]. The specific chemical structure of catechins has a significant impact on the antioxidant capacity [13]. Chemical structure of catechins include an aromatic ring A that structurally resembles a resorcinol molecule, a second aromatic ring B connected to ring C that structurally resembles pyrocatechol (catechol) or pyrogallol, depending on the number of hydroxyl groups, and a heterocyclic dihydropyran ring C that links rings A and B. Ring C contains a hydroxyl group at the C3 carbon and two chiral centers (C2 and C3), which determine the specific form of the molecule (e.g., catechin vs. epicatechin). Moreover, esterified catechins, such as epicatechin gallate (ECG) and epigallocatechin gallate (EGCG), contain ring D with galloyl group (Figure 1). Catechins also are able to chelate off transition metal ions due to the di/tri-hydroxy structure of the B and D rings [12], as well as the meta–5,7–dihydroxy group at the A ring [14].

Various model systems have been applied to the characterization of the multifunctional activity of catechins [15]. In this paper, the term “multifaceted biological activity” can be used in a broader aspect when the inhibition of the activity of a key enzyme (IC_50_) important in diabetic prophylactic activity (in vitro inhibitory effect on alpha-amylase and alpha-glucuronidase activity), obesity prophylactic activity (in vitro inhibitory effect on pancreatic lipase activity), against neurological disorders (inhibition of acetylcholinesterase activity—important for treatment of Alzheimer-type dementia), against hypertension (inhibition of angiotensin-I-converting enzyme activity), against glycation important in diabetic complications (inhibition of advanced glycation product formation and against inflammation (inhibition of COX-1 activity) is addressed. In this revised manuscript, the term “multifaceted activity” is related to their antioxidant activity, inhibition of angiotensin-converting enzyme (ACE) activity, inhibition of acetylcholinesterase (AChE) activity, and inhibition of advanced glycation end-product (AGE) formation to show their importance against hypertension, Alzheimer-type dementia, and diabetic complications, respectively. The antioxidant activity of catechins was characterized by their oxidation potential, and searching for their correlation with other biological activities may suggest that the inhibitory activity against ACE, AChE activity, and AGE formation may occur with the free radical scavenging effect, which is not exactly connected to the formation of reactive oxygen or nitrogen species. The electron-donating groups (measured by voltammetry) may facilitate binding to specific catalytic pockets or modulate enzymatic redox states. Moreover, docking studies indicated that phenolic compounds inhibit ACE via interaction with the zinc ion, and this interaction is stabilized by other interactions with amino acids in the active site [16,17,18,19].

Electrochemical methods such as cyclic voltammetry (CV), differential pulse voltammetry (DPV), and square wave voltammetry (SWV) for determination of the antioxidant activity have emerged in the past two decades [20,21,22,23,24]. Usually, voltammetric data provide qualitative information (peak of anodic oxidation potential (E_pa_) indicating antioxidant strength) and quantitative information (peak currents or charges calculated in predefined potential ranges) proportional to antioxidative activity. The low value of E_pa_ corresponds to strong antioxidant power [16,25]; therefore, DPV is an excellent technique to determine the E_pa_.

Hypertension seriously affects the world’s adult population and it is a risk factor to the development of cardiovascular disease (CVD) [26,27]. Hypertension is often treated with synthetic drugs that inhibit angiotensin-I-converting enzymes [28]. However, their action is often accompanied with unbeneficial side effects such as skin rashes, dry cough, hypotension, and angioedema [29]. For this reason, searching for alternative natural inhibitors is of great interest, and screening of the ACE inhibitory activities of catechins may provide new knowledge on the verification of their functional properties.

Alzheimer’s disease (AD) and related neurological disorders are characterized clinically by the decreased level of the neurotransmitter acetylcholine (ACh) observed in the brains of the elderly population, resulting in a loss of cognitive ability, severe behavioral abnormalities, and ultimately death [30,31,32,33]. Therefore, acetylocholine esterase inhibitors are currently the only approved therapy for the treatment of AD, increasing the amount of ACh present in the synapses between cholinergic neurons [34,35]. Natural phytochemicals seem to be a good option for AChE inhibition, and compounds such as alkaloids, flavonoids, and phenolics represent an interesting class of biologically active inhibitors [36,37,38]. The number of new flavonoids with acetylinesterase inhibitory effects is increasing. In 2006, there were only 14 flavonoids reported [39], while in 2011, 128 flavonids were classified as AChE inhibitors [35].

Advanced glycation end-product (AGE) formation with aging is a risk factor for diabetes, uremia, cataract, atherosclerosis, and Alzheimer’s disease [40,41]. Dietary AGEs are the most important pro-inflammatory compounds originating from the Maillard reaction, and they have nutritional and toxicological effects on processed food and in turn for consumers [42]. There is a basic difference between dietary AGEs from Maillard reaction and those generated endogenously in human organisms. Endogenous AGEs are formed from reducing sugars and proteins, independent of their structure. Different inhibitors suppressing AGE formation are presented in food. Various phenolic antioxidants from plants and food of plant origin have been found to inhibit the formation of AGEs, and the inhibition of free radical generation in the glycation process has been considered as the major mechanism of their anti-AGE glycation mediation [43,44,45].

The aim of the study was to demonstrate the multifaceted activity of catechins: (+)-catechin (**C**), (-)-epicatechin (**EC**), (-)-epigallocatechin (**EGC**), (-)-epicatechin-3-gallate (**ECG**), and (-)-epigallocatechin-3-gallate (**EGCG**) (Figure 1). The antioxidant activity was determined by the DPV technique, whereas the ability of these compounds to inhibit ACE activity, AChE activity, and AGE formation was performed in a model system to show their importance against hypertension, Alzheimer-type dementia, and diabetic complications, respectively. Then, the relationship between the multifaceted activity of these compounds and their antioxidant activity provided by the DPV technique was addressed.

## 2. Results and Discussion

### 2.1. The Antioxidant Potency of Catechins Provided by the Voltammetric Experiments

**Figure 2:**
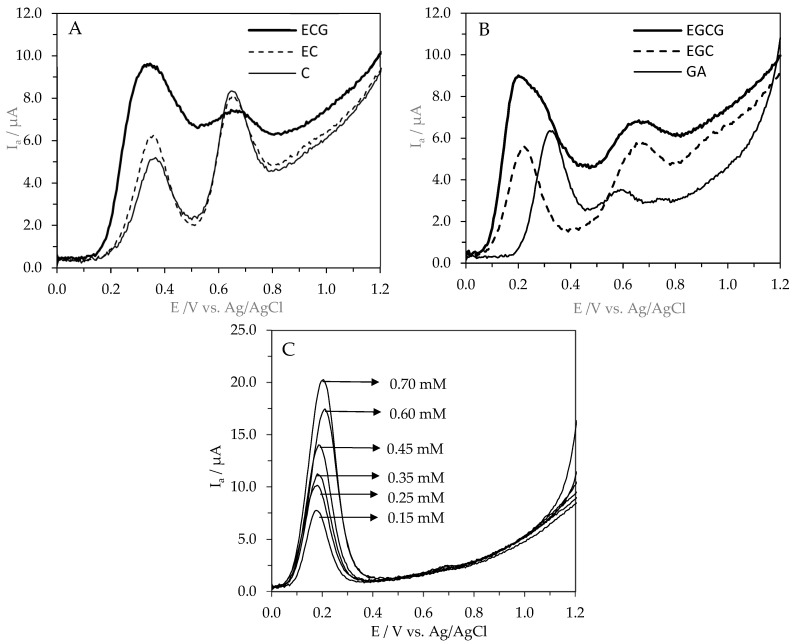
Differential pulse voltammograms of 0.25 mM standard solutions of (**A**) C, EC, and ECG; (**B**) GA, EGC, and EGCG; and (**C**) Trolox solutions in the concentration range from 0.15 to 0.70 mM.

In this study, the antioxidant potential of (+)-catechin (**C**), (-)-epicatechin (**EC**), (-)-epigallocatechin (**EGC**), (-)-epicatechin-3-gallate (**ECG**), and (-)-epigallocatechin-3-gallate (**EGCG**) was described by the first (E_pa1_) and second (E_pa2_) anodic oxidation potentials and the intensity of the anodic current (I_a_) in comparison to gallic acid (GA) and Trolox. The oxidation of catechins is of great interest because they act as antioxidants scavenging free radicals through the electron transfer process [46]. To determine the antioxidant potential of these compounds, the area under the AC wave was used as previously suggested [47]. The recordered cyclic voltammograms are shown on Figure 2.

**C**, **EC**, **EGC**, **ECG**, **EGCG**, and **GA** showed well-defined oxidation peaks with the first peak potentials of 380, 334, 220, 341, 251, and 320 mV (vs. Ag/AgCl) for 0.25 mM standard solutions in 0.1 M acetate–acetic buffer (pH 5.0) in 80% methanol (Table 1).

In this study, the order of the antioxidant potential of (+)-catechin (**C**), (-)-epicatechin (**EC**), (-)-epigallocatechin (**EGC**), (-)-epicatechin-3-gallate (**ECG**), (-)-epigallocatechin-3-gallate (**EGCG**), and gallic acid (**GA**) was **EGCG** > **ECG** > **EC** > **EGC** ≈ **C** > **GA**. The antioxidant activity of **EGCG**, **ECG**, **EC**, **EGC**, and **C** was higher by 64, 60, 45, 20, and 19% compared to the antioxidant activity of **GA**. The antioxidant activity of **EGCG** and ECG was significantly higher than the antioxidant activity of **C**, **EC**, **EGC**, and **GA** (*p* ≤ 0.05). This reflects the specific chemical structure of catechins (the presence of a minimum of five hydroxyl groups), especially the di/tri-hydroxy structure of the B and D rings [13], as well as the meta-5,7-dihydroxy group at the A ring [13,14]. In addition to the number of hydroxyl groups, their distribution is important. Catechins with a catechol group (**C**, **EC**, **ECG**) have lower antioxidant potential compared to catechins with a pyrogalol group. However, the antioxidant efficacy of catechins depends not only on the chemical structure, but also on the environmental conditions [5]. Wang et al. [48] showed that antioxidant activities of catechins were dominated by B-ring pyrogallol and 3-galloyl, but were not decided by geometrical isomerism.

The number, not the position, of the galloyl group was positively correlated with the antioxidant activities of theaflavins [48]. Moreover, taking into account the values of the first oxidation potential (E_pa1_) of the studied compounds, **EGC** and **EGCG** can be classified as compounds with high antioxidant power (Ep < 0.3 V), whilst **EC**, **ECG**, and **C** can be classified as compounds with intermediate potential (0.3 V < Ep < 0.8 V). This conclusion was withdrawn according to the work by Blasco et al. [49], in which differentiation of the antioxidant power of phenolic compounds was based on the values of their oxidation potential. Moreover, the antioxidant activity of catechins provided by DPV were well correlated with the Ferric Reducing Antioxidant Power (FRAP) described by Grzesik et al. [46], and the correlation coefficient had the value r = 0.86. The provided data were also in accordance with the standard reduction potential of these compounds (E^0^), which describes the ability of a compound to accept electrons. Baranowska et al. [15], using the potentiometric titration performed vs. 3 M KCl Ag/AgCl reference electrode and a platinum measuring electrode, showed the reduction potential of catechins. The standard reduction potential for catechins showed the order **ECG** (146 mV) ˂ **EGCG** (153 mV) ˂ **EGC** (287 mV) ˂ **C** (281 mV) ˂ **EC** (277 mV). The lower the value of the standard reduction potential of a compound, the better an electron donor it is, which means that the compound exhibits stronger antioxidant properties. In our study, the highest antioxidant activity of **ECG** and **EGCG** was in agreement with their standard reduction potential, while the activity of **EGC**, **EC**, and **C** were on a comparable level. This comparison clearly indicates that the standard reduction potentials of catechins, the values of the first (E_pa1_) and second (E_pa2_) anodic oxidation potentials, and the intensity of the anodic current (I_a_) provided by DPV technique depended not only on the number of hydroxyl groups, but also on the site of substitution (Figure 1). Among catechins, the parent structure of **C** displayed the lowest antioxidant activity, and the introduction of a hydroxyl group at position 3′ (**EGC**) did not improve antioxidant properties (Table 1). In contrast to the data provided by Baranowska et al. [15], the stereoisomer **EC** exhibited higher antioxidant activity than **C**. The esterification of a hydroxyl group at position 3 with gallic acid markedly increased the antioxidant activity of both **ECG** and **EGCG**, the latter becoming the strongest antioxidant among the catechins tested (Table 1). This conclusion was also in agreement with the early measurement of the oxidation potential of catechins by employing flow-through column electrolysis, which showed stronger antioxidant activity of galloylated catechins than those of nongalloylated catechins [50]. Moreover, Masek et al. [51], using cyclic (CV) and differential pulse (DPV) voltammetry methods, showed mechanism of the electrochemical oxidation of catechin at a platinum (Pt). The structure of catechin has OH groups attached to the rings, which can be electrochemically oxidized. As determined by the distribution of electron charges in the molecule, the oxidation group OH in ring B was the most easily oxidized. Based on the experimental results and quantum chemistry calculations, the first step of the electrooxidation process of catechin includes the exchange of one electron and two protons, resulting in the formation of a semiquinone. The next step is the exchange of a second electron, resulting in the formation a quinone [51]. With all these evidence, the question regarding the impact of the antioxidant potential of **C**, **EC**, **EGC**, **ECG**, and **EGCG** on their multifaceted biological activities can be addressed.

### 2.2. Angiotensin-I-Converting Enzyme Inhibitory Activity of Catechins

Angiotensin-I-converting enzyme (ACE) plays a key physiological role in the control of blood pressure in the renin–angiotensin system. ACE converts the inactive decapeptide, angiotensin I, into a potent vasopressor octapeptide, angiotensin II, and inactivates bradykinin [52]. Therefore, the influences of ACE on blood pressure make it an ideal target, both clinically and nutritionally, in the treatment of hypertension. Elevated blood pressure is often treated with synthetic drugs that inhibit ACE, such as captopril and enalapril, but synthetic ACE inhibitors might be accompanied by side effects [26]. Therefore, the need for alternative natural inhibitors is of great interest. The ACE inhibitory activity of catechins is shown in Table 2.

The IC_50_ values of catechins for angiotensin-I-converting enzyme inhibition ranged from 43.4 µM to 8279.3 µM in comparison to glutathione (IC_50_ = 41.7 µM) and captopril (IC_50_ = 0.0059 µM). The IC_50_ value represents the concentration of each compound that inhibits ACE activity by 50%. Lower IC_50_ values and higher ACE inhibitory activity were noted. The IC_50_ values of catechins were significantly different (*p* < 0.05) based on the one-way analysis of variance (ANOVA). The order of the ACE inhibitory activity of catechins based on their IC_50_ values was **EGCG** > **ECG** > **EGC** > **EC** > **C** (Table 2), thus indicating the importance of the structure–activity relationship. In this study, a negative correlation was found between IC_50_ of catechins and their antioxidant activity provided by the DPV technique. The correlation coefficient had the value r = −0.60, thus clearly indicating the impact of the antioxidant potential and chemical structure on the ACE inhibitory activity of **C**, **EC**, **EGC**, **ECG** and **EGCG**. It can be concluded that the higher the antioxidant potential of the compounds, the higher the ACE inhibitory activity observed. This finding was also confirmed by the positively correlation found between ACE inhibitory activity of catechins (IC_50_) and their first anodic peak potential (E_pa1_) values (r = 0.70). A similar value of correlation coefficient between the ACE inhibitory activities of quercetin and their glucosides and the antioxidant potential provided by the DPV technique was recently reported by our team (r = 0.68) [16]. In this study, the highest ACE inhibitory activity of **EGCG** (IC_50_ = 43.36 µM) was comparable to the ACE inhibitory activity of quercetin (IC_50_ = 64 µM), quercetin rutinoside (IC_50_ = 43 µM) [15], luteolin (IC_50_ = 77 µM), luteolin-7-*O*-glucoside (IC_50_ = 62 µM), and kaempferol-7-*O*-glucoside (IC_50_ = 98 µM) [17]. On the other hand, the ACE inhibitory activity of **EGCG** was about 3, 6, 50, and 192 times higher than the activity of **ECG**, **EGC**, **EC**, and **C**. However, the ACE inhibitory activity of **EGCG** was generally low compared to captopril, {1-(3-mercapto-2-D-methyl-1-oxopropyl)-L-proline}, which is used therapeutically as an antihypertensive drug that treats high blood pressure, heart failure, and heart damage after a heart attack [53].

Recently, a lot of different plant-based foods, along with several phytochemicals such as quercetin, glycosides of quercetin, apigenin, cyanidin, kaempferol, and luteolin, were reported to inhibit ACE [54,55]. Moreover, Zielińska et al. [17] showed a moderate ACE inhibitory activity of low-molecular-weight phenolic metabolites of flavonoids and the high ACE inhibitory activity of flavonoid aglycones such as luteolin, quercetin, kaempferol, cyaniding, delphinidin, pelargonin, and naringenin, while a lower inhibition activity was noted for their 3-*O*-glucosides and 7-*O*-glucosides. These findings, following other observations, noted that the position and number of hydroxyl groups in the catechin’s structure as well as in other polyphenols significantly affect their ACEinhibitory activity [18,19,51,55,56,57]. The provided ACE inhibitory activity of **EGCG** was comparable with that of **GSH**, thus indicating the importance of both sources of glutathione as well as other peptides in increased blood pressure treatment by dietary intervention or enhanced drug therapy.

### 2.3. Acetylcholinesterase Inhibitory Activity of Catechins

Acetylcholinesterase (AChE) inhibitors are used for the treatment of Alzheimer’s disease (AD), increasing the neurotransmitter acetylcholine levels at cerebral cortex synapses [30,32,36]. The AchE inhibitory activity of catechins is shown in Table 3.

The IC_50_ values of catechins for AChE inhibition ranged from 1.589 µM to 19.651 µM in comparison to galanthamine (IC_50_ = 0.0437 µM). The IC_50_ value represents the concentration of each compound that inhibits AChE activity by 50%. Lower IC_50_ values followed by higher AchE inhibitory activity were noted. The IC_50_ values of catechins were significantly different (*p* < 0.05) based on the one-way analysis of variance (ANOVA) (Table 3). **EGC** and **EGCG** showed the highest AchE inhibitory activity; however, their IC_50_ values were about 33–37 times lower as compared to the activity of galantamine. The lowest IC_50_ values were noted for **C** and **EC**, and these values were statistically significantly different as compared to the remaining compounds. The order of the AChE inhibitory activity was **galantamine** > **EGC** ≈ **EGCG** > **ECG** > **EC** > **C**, thus indicating a relationship with the chemical structure. Galantamine, originally isolated from plants of the Amaryllidaceae family [58] and from snowdrop [59], has become an important treatment for AD. The AchE inhibitory activity of this drug is the principal mode of action to provide symptomatic relief. It is licensed in Europe for AD treatment and was well tolerated and significantly improved cognitive function when administered to AD patients in multi-center randomized-controlled trials [59]. As **EGC** and **EGCG** were classified as compounds with a high antioxidant power (Ep < 0.3 V), whilst **EC**, **ECG**, and **C** were classified as compounds with an intermediate potential (0.3 V < Ep < 0.8 V), a correlation between their first anodic peak potential (E_pa1_) values provided by the DPV technique and AchE inhibitory activities was investigated. The correlation coefficient had the value r = 0.67, thus indicating a relationship between AchE-inhibitory activity and the chemical structure of catechins. It means that the lower the first anodic peak potential of catechins, the lower the IC_50_ value, indicating a stronger AchE inhibitory activity. A similar value of correlation coefficient between AchE inhibitory activities of quercetin and their glucosides and the antioxidant potentials provided by the DPV technique was recently reported by Zielińska et al. [16]. In contrast, no correlation between AchE inhibitory activity and the values of E_pa_ and antioxidant activity of selected flavone *C*-monoglucosides was found [60].

In this study, the strong AchE inhibitory activities of **EGC** and **EGCG** were comparable to the AchE inhibitory activities of quercetin and its glucosides, quercetin rutinoside, and selected flavone *C*-monoglucosides such as vitexin and isovitexin, while other flavone *C*-monoglucosides such orientin and homoorrientin showed two-to-threefold higher inhibitory activity [16,60]. On the other hand, the AchE inhibitory activity of **EGC** was only higher by 8% than the activity of **EGCG**, about twice as high as the inhibitory activity of **ECG** and almost 10 and 14 times stronger in comparison to the inhibitory activity of **C** and **EC** (Table 3).

Acetylocholine esterase inhibitors are currently the only approved therapy for the treatment of Alzheimer’s diseases, increasing the amount of ACh present in the synapses between cholinergic neurons [34]. Over the last decade, the alkylpyridium polymers, dehydroevodiamine (DHED), and carbamate-type AChE inhibitors have been reported, but because of bioavailability problems and possible side effects, there is still great interest in finding better AChE inhibitors [31,61]. The current study showed strong AChE inhibitory activity of **EGC** and **EGCG**. Therefore, unfermented green tea, grape, and wine may serve as natural sources of these compounds [62]. In a study performed by Okello et al. [33], acetylcholinesterase inhibition by white and green teas and their simulated intestinal digests were provided. These beneficial properties were largely attributed to high polyphenol content, particularly the catechins. Of the pure tea compounds tested, **EGCG** was the most active against AChE inhibition. In general, **EGCG** and **ECG** promote lifespan, fitness, and stress resistance when applied at low doses [63]. These results were in accordance with the recently reported strong inhibitory activity of **EGCG** and **EGC** [64], indicating the importance of unfermented green tea catechins as attractive inhibitors of AChE. Further in vivo testing of the most active **EGCG** is recommended to fully characterize its multifunctional properties.

### 2.4. The Inhibitory Activity of Catechins Against Advanced Glycation End-Product (AGE) Formation

The multifaceted biological activity of catechins also covers the inhibition of advanced glycation end-product (AGEs) formation, which were proposed to be causative factors for various kinds of diseases, especially diabetes [39,40]. The inhibitory effect of standard solutions of catechin (**C**), epicatechin (**EC**), epigallocatechin (**EGC**), epicatechin-3-gallate (**ECG**), and epigallocatechin-3-gallate (**EGCG**) in comparison to aminoquanidine (**AG**) was determined by BSA/glucose (bovine serum albumin/glucose) and BSA/MGO (bovine serum albumin/methylglyoxal) model systems. The inhibitory activity of catechins against AGE formation is shown in Table 4.

Catechins at a concentration of 1 mM demonstrated a high inhibitory activity against AGE formation (74–92%) in the BSA–glucose model and 59–90% in the BSA–MGO model, compared to 1 mM aminoguanidine (81% and 79% inhibition, respectively). A linear dependence was observed between the concentration of catechins and the percentage of inhibition (R^2^ = 0.96). Based on this, IC_50_ values were determined, which allowed the ranking of the anti-AGEs activities in the BSA-glucose model (**EGCG** ≈ **ECG** > **EGC** > **EC** ≈ **AG** > **C**) and in the BSA/MGO model (**EGCG** > **EGC** > **ECG** ≈ **EC** ≈ **AG** > **C**). The IC_50_ values against AGE formation in the BSA/glucose model system ranged from 0.331 to 0.499 mM as compared to aminoquanidine (IC_50_ = 0.436 mM). **EGCG** was the strongest inhibitor of AGE formation. The anti-AGE activity of **EGCG** was higher by 8, 22, 28, 51, and 32% in comparison to **EGC**, **ECG**, **EC**, **C**, and **AG**, and the same trend was noted for these compounds in BSA/MGO model system. The strong AGE inhibitory activity of **EGCG** was comparable to the AGE inhibitory activity of quercetin and its glucosides, quercetin rutinoside, and selected flavone *C*-monoglucosides such as vitexin and isovitexin, while other flavone *C*-monoglucosides such orientin and homoorrientin showed two-to-threefold higher inhibitory activity [16,60].

A negative correlation between the inhibitory activity of catechins against AGE formation in the BSA/glucose model system and their antioxidant activity provided by the DPV technique had the value r = −0.82, thus indicating the relationship between anti-AGE activities and the chemical structure of catechins. This finding was supported by the positive correlation noted between the inhibitory activity of catechins in the BSA/MGO model system and their first anodic peak potential (E_pa1_) values (r = 0.88). It means that the lower the first anodic peak potentials of catechins, the lower the IC_50_ values, indicating stronger inhibitory activity against AGE formation. Moreover, IC_50_ of catechins obtained in BSA/glucose and BSA/MGO model systems were weakly positively correlated with their IC_50_ values for AChE inhibition (r = 0.68 and r = 0.64), whilst a stronger correlation was noted for ACE inhibition (r = 0.90 and r = 0.92).

In recent decades, phytochemicals play an essential role in medicine, since the need to investigate highly effective and safe drugs for the treatment of diabetes mellitus disease remains a significant challenge for modern medicine [65]. Our results clearly indicate that **EGCG** displays the strongest inhibitory capacity on AGE formation as compared to aminoquanidine. The results provided in this study support the available data on the anti-diabetic, anti-obesity, and hypotensive effects of catechins [12,13].

## 3. Materials and Methods

### 3.1. Chemicals

The following compounds were used for the study: (+)-catechin (**C**), (-)-epicatechin (**EC**), (-)-epigallocatechin (**EGC**), (-)-epicatechin-3-gallate (**ECG**), and (-)-epigallocatechin-3-gallate (**EGCG**) from Extrasynthese (Genay, France). Methanol, acetic acid (supra-gradient), and sodium acetate were from Merck KGaA, Darmstadt, Germany. Trolox (6-hydroxy-2,5,7,8-tetramethylchroman-2-carboxylic acid, 2,4,6-tris(pyridyl-s-triazine) (TPTZ), glutathione (GSH), sodium azide, bovine serum albumin (BSA), D-glucose, methyl glioxal (MGO), and aminoguanidine hydrochloride were purchased from Sigma (Sigma Chemical Company, Saint Louis, MO, USA). Angiotensin-converting enzymes (ACEs) from porcine kidneys (EC 3.4.15.1) were purchased from Sigma-Aldrich (St. Louis, MO, USA). The substrate *o*-aminobenzoylglycyl-*p*-nitorphenylalanylproline (Abz-Gly-Phe(NO_2_)-Pro) was obtained from BACHEM (Bubendorf, Switzerland). Diethyl ether (Et_2_O), hydrochloric acid (HCl), and sodium hydroxide (NaOH) were obtained from Avantor Performance Materials Poland S.A. (Gliwice, Poland). Captopril was obtained from Sigma-Aldrich (No C4042, St. Louis, MO, USA). Acetylthiocholine iodide (ATCI), acetylcholinesterase (AChE type VI-S, from *Electrophorus electricus*), 5,5′[2-nitrobenzoic acid] (DTNB), and galanthamine hydrobromide were obtained from Sigma Chemical Co. (Poznań, Poland). All other reagents of reagent-grade quality were from POCh, Gliwice, Poland. Water was purified with a Mili-Q-system (Milipore, Bedford, MA, USA).

### 3.2. Measurement of the Anodic Oxidation Potentials of Catechins with Differential Pulse Voltammetry

The 1 mM concentration of **C**, **EC**, **EGC**, **ECG**, and **EGCG** in 80% methanol (1 mM) was prepared. Electrochemical measurements were performed at room temperature (25 ± 2 °C) in a small-volume electrochemical cell using a potentiostat-type SP-240 (BioLogic Science Instruments, Seyssinet-Pariset, France) controlled by EC-Lab V11.36 software, as described recently [60]. Differential pulse voltammograms were obtained with a pulse amplitude of 100 mV, a potential step of 5 mV, and a modulation time of 0.2 s. Four scans were registered for each sample, and the reported data correspond to the average of at least three replicates. The working electrode was a glassy carbon electrode, GCE (3 mm diameter, BASi M-2012), and the auxiliary and reference electrodes were platinum wire (BASi MW-1033) and Ag/AgCl (3 M KCl, BASi-MW-2052). All potentials are quoted against the used reference electrode. Before each scan, the surface of the glassy carbon electrode was polished on a polishing cloth with 0.05 μm alumina paste and ultrasonically rinsed in deionized water. After polishing, the electrode was washed with ultrapure water and dried with absorbent paper. Before measurements, the standard solutions were diluted in 0.2 M acetate–acetic buffer (in 80% methanol) at pH 5.0 (*v*/*v*: 1:1). The DPV measurements were carried out in a standard solution of catechins at a final concentration of 250 μM. Buffer solution also served as a supporting electrolyte [15,16,18,59]. For testing purposes, from registered DPV voltammograms, the anodic oxidation potentials (E_pa_) in mV and the integrated area under anodic peaks in μA were determined. The same apparatus and conditions were also used for voltammetric experiments with Trolox solutions (Trolox concentration ranged from 0.15 to 0.70 mM) mixed with 0.2 M sodium acetate–acetic buffer (pH 4.5 in 80% methanol) at a ratio of 1:1 (*v*/*v*)]. Based on the area under the anodic current (AC) waves of Trolox solutions, the equation of the linear regression (y = 133.29x + 8.21; R^2^ = 0.99) was used for the calculation of antioxidant activity of EGCG, ECG, EC, EGC, C, and GA expressed as Trolox equivalent (TE) in Table 1.

### 3.3. Angiotensin-I-Converting Enzyme Inhibitory Assay

Standards of catechins were dissolved in 80% methanol. Each standard was diluted to various concentrations, using deionized water to determine its ACE inhibitory activity, and expressed as IC_50_ value. ACE stock solution was prepared by dissolving enzyme in glycerol at 50% with buffer A (protein concentration should be approximately 150 μg/mL), whereas the working solution was prepared by dilution of the stock solution with buffer B to make a protein concentration of approximately 7.5 μg/mL. Substrate stock solution was prepared by dissolving the whole content (50 mg) of Abz-Gly-Phe (NO_2_)-Pro (Bachem) in 10.33 mL of buffer C (concentration approximately 10 mM), while a working solution was done by diluting substrate stock solution with buffer C to make a final Abz-Gly-Phe (NO_2_)-Pro concentration of approximately 0.45 mM. Captopril solution 0.1 μM was prepared in deionized water.

Angiotensin-converting enzyme (ACE) inhibitory assay was performed according to the method of Sentandreu and Toldra [66]. The procedure was based on the hydrolysis of the internally quenched substrate *o*-aminobenzoylglycyl-*p*-nitorphenylalanylproline (Abz-Gly-Phe(NO_2_)-Pro) in accordance with the reaction of ACE. The fluorescence generated by the liberation of the product (the *o*-aminobenzoylglycine group) was measured immediately after mixing (0 min). Then, after 30 min of reaction, a multiscan microplate fluorometer at an excitation of 365 nm and an emission of 405 nm (Tecan Infinite M1000 PRO, TK Biotech, Warsaw, Poland) was used. To calculate the % of inhibition of ACE, the following equation was used:

Relative ACE activity % = 100 − (ΔRFU_sample_ × 100/ΔRFU_negative control_)

where ΔRFU = RFU_at time 30_ − RFU_at time 0._

The IC_50_ value indicating the sample concentration of 50% inhibition of ACE activity was determined by using linear regression analysis of logarithmic plots. At least 3 replicates for each standard solution were conducted. Results were expressed as IC_50_ values.

### 3.4. Acetylcholinesterase Inhibitory Assay

The AChE inhibitory activity of pure standard compounds was evaluated following the methodology adapted from Eldeen et al. [67]. Three buffers were prepared: Buffer A (50 mM Tris–HCl, pH 8.0), Buffer B (50 mM Tris–HCl, pH 8.0, containing 0.1% bovine serum albumin), and Buffer C (50 mM Tris–HCl, pH 8.0, containing 0.1 M NaCl and 0.02 M MgCl_2_·6H_2_O). In a 96-well microplate, the following components were combined: 25 μL of 15 mM acetylthiocholine iodide (ATCI) dissolved in water, 125 μL of 3 mM 5,5′-dithiobis(2-nitrobenzoic acid) (DTNB) prepared in Buffer C, 50 μL of Buffer B, and 25 μL of the test compound solution at concentrations ranging from 0.01 to 1 mg/mL. Galanthamine (0.5–50 μg/mL in water) served as the positive control, while water was used as the negative control. Initial absorbance readings at 405 nm were recorded every 45 s for a total of five measurements to establish baseline activity. Subsequently, 25 μL of AChE solution (0.2 U/mL in Buffer B) was added to each well, and absorbance was measured eight times at 45-s intervals to monitor enzymatic activity. All measurements were conducted using a microplate reader (Tecan Infinite M1000 PRO, TK Biotech, Warsaw, Poland). Each assay was performed in triplicate to ensure reproducibility. The inhibition of AChE activity was calculated as follows:

$$AChE\;inhibition\;\left(\%\right)=\;100-(∆{Abs}_{sample}\times 100/{Abs}_{negative\;control})$$

where ΔAbs (Absorbance) = Abs_with enzyme after 360s_ − Abs_without enzyme 225s_.

The IC_50_ indicating the concentration of the compound, at which AChE activity is inhibited by 50%, was determined through linear regression analysis, with a coefficient of determination (R^2^) ranging from 0.805 to 0.999, based on triplicate measurements.

### 3.5. Inhibition of the Formation of Advanced Glycation End-Products (AGEs)

The inhibitory activity against AGE formation was performed in the bovine serum albumin (BSA)/glucose and BSA/methylglioxal (MGO) model systems as described recently by Zielińska et al. [16]. The BSA–glucose model adopted in this study provides a useful tool for assessing the effects of catechins on the non-enzymatic glycation process. Generally, there are three stages in the non-enzymatic glycation process in vivo. First, glycation is initiated by the covalent attachment of reducing sugars to amino groups of proteins, lipids, or nucleic acids to produce a reversible and unstable Schiff base. Then, the Schiff base may undergo Amadori rearrangement and change to a more stable Amadori product. Subsequently, Amadori products undergo dehydration and rearrangement to form highly reactive carbonyl species including 3-deoxy glucosone (3-DG), glyoxal (GO) and methylglyoxal (MGO). Finally, reactions between these reactive carbonyls and amino, sulphydryl, and guanidine functional groups of intracellular and extracellular proteins would result in the formation of AGEs. It was reported that some tea polyphenols could trap MGO under physiological conditions [68]. Therefore, to examine whether catechins are effective in inhibiting direct MGO-induced AGE formation, the BSA/MGO model system was used in our study.

Standards of catechins were initially dissolved in a small volume of DMSO and then in phosphate buffer (0.1 M, pH 7.4) (DMSO/phosphate buffer; 1:5; *v*/*v*) to obtain 1 mM solutions. The positive control aminoguanidine (1 mM AG) was used [69]. The percentage (%) of inhibition was obtained from three repetitions (n = 3). The IC_50_ indicating the concentration of the compound, at which formation of AGEs is inhibited by 50%, was determined through linear regression analysis (Figure S1).

### 3.6. Statistical Analysis

Results are given as the average ± standard deviation (SD). The one-way analysis of variance (ANOVA) was used for the analysis of significant differences in the multifaceted biological activities of catechins (*p* < 0.05) (GraphPad Prism version 9 for Windows, GraphPad Software, San Diego, CA, USA). The correlation analysis was performed and the Pearson correlation coefficient was calculated.

## 4. Conclusions

The **EGCG** and **ECG** showed high ACE and AChE inhibitory activity, whereas all tested catechins were strong inhibitors of AGE formation. The order of the antioxidant potential of catechins in comparison to gallic acid (GA) was **EGCG** > **ECG** > **EC** > **EGC** ≈ **C** > **GA**, whereas the order of the ACE inhibitory activity was **EGCG** > **ECG** > **EGC** > **EC** > **C**. The order of the AChE inhibitory activity was **EGCG** ≈ **EGC** > **ECG** > **EC** > **C**, while the rank of the anti-AGE activities was **EGCG** ≈ **ECG** > **EGC** > **EC** > **C** in both model systems. The correlation between IC_50_ for ACE inhibition of catechins and their antioxidant activity provided by the DPV had the value r = −0.60, thus indicating the importance of the structure–activity relationship. The weak positive correlation between IC_50_ for AChE inhibition and the first anodic peak potential (E_pa1_) values was noted (r = 0.67). A negative correlation between the inhibitory activity of catechins against AGE formation in the BSA/glucose model system and their antioxidant activity provided by the DPV technique had the value r = −0.82, thus indicating the relationship between anti-AGE activity and the chemical structure of catechins. This finding was supported by the positive correlation noted between the inhibitory activity of catechins in the BSA/MGO model system and their first anodic peak potential (E_pa1_) values (r = 0.88). The provided results expand our knowledge on the multifaceted activity of catechins, indicating **EGCG** and **ECG** as the most active antioxidants against inhibition of ACE and AChE as well as towards AGE formation.

## Figures and Tables

**Table 1:** The anodic peak potentials (*E_pa_*) and antioxidant activity of catechins in comparison to gallic acid (GA) provided by differential pulse voltammetry technique.

Compound	Anodic Peak Potential *E_pa_* (mV)		Antioxidant Activity (mM Trolox)
	*E*_*pa*1_	*E*_*pa*2_	
**C**	380 ± 5 ^a^	655 ± 1 ^bc^	1.18 ± 0.04 ^c^
**EC**	334 ± 4 ^bc^	647 ± 7 ^cd^	1.44 ± 0.06 ^a^
**EGC**	220 ± 9 ^e^	662 ± 5 ^ab^	1.19 ± 0.05 ^c^
**ECG**	341 ± 7 ^b^	655 ± 1 ^c^	1.58 ± 0.06 ^a^
**EGCG**	251 ± 9 ^d^	657 ± 5 ^bc^	1.62 ± 0.06 ^ab^
**GA**	320 ± 4 ^c^	590 ± 1 ^e^	0.99 ± 0.04 ^d^

The antioxidant activity of standard solutions of (+)-catechin (**C**), (-)-epicatechin (**EC**), (-)-epigallocatechin (**EGC**), (-)-epicatechin-3-gallate (**ECG**), and (-)-epigallocatechin-3-gallate (**EGCG**) in comparison to gallic acid (**GA**) was determined within the concentration range of 1.0–0.01 mM. Data are expressed as means ± standard deviation (n = 3). Equation of the linear regression of Trolox concentrations (y = 133.29x + 8.21; R^2^ = 0.99) was used for the calculation of antioxidant activity. Different superscript letters (a–e) within a column indicate significant differences at *p* < 0.05.

**Table 2:** The IC_50_ of catechins for ACE inhibition (µM).

Compound	Equation of the Linear Regression	IC_50_ (µM)
**C**	y = 0.0029x + 25.99 R^2^ = 0.96	8279.31 ± 142.31 ^a^
**EC**	y = 0.0139x + 20.22 R^2^ = 0.98	2142.45 ± 13.27 ^b^
**EGC**	y = 0.1416x + 14.811 R^2^ = 0.94	248.52 ± 9.27 ^c^
**ECG**	y = 0.2416x + 17.354 R^2^ = 0.94	135.12 ± 1.57 ^c^
**EGCG**	y = 0.4178x + 31.879 R^2^ = 0.94	43.36 ± 1.13 ^d^
**GSH**	y = 0.1337x + 44.429 R^2^ = 0.98	41.68 ± 3.45 ^d^
**Captopril**	y = 5008.1x + 20.632 R^2^ = 0.98	0.00586 ± 0.00004 ^e^

The inhibitory effect of standard solution of (+)-catechin (**C**), (-)-epicatechin (**EC**), (-)-epigallocatechin (**EGC**), (-)-epicatechin-3-gallate (**ECG**), and (-)-epigallocatechin-3-gallate (**EGCG**) in comparison to reduced glutathione (**GSH**) and captopril was determined within the concentration range of 1.0–0.01 mM. Data are expressed as means ± standard deviation (n = 6). Equation of the linear regression was used for IC_50_ calculation. Different superscript letters (a–e) within a column indicate significant differences at *p* < 0.05.

**Table 3:** The IC_50_ of catechins for acetylcholinesterase (AChE) inhibition (µM).

Compound	Equation of the Linear Regression	IC_50_ (µM)
**C**	y = 3.037x + 8.773 R^2^ = 0.99	13.575 ± 0.504 ^a^
**EC**	y = 8.305x + 2.626 R^2^ = 0.99	19.651 ± 1.243 ^a^
**EGC**	y = 16.708x + 26.25 R^2^ = 0.97	1.421 ± 0.030 ^c^
**ECG**	y = 6.723x + 29.176 R^2^ = 0.98	3.098 ± 0.257 ^b^
**EGCG**	y = 31.654x − 0.291 R^2^ = 0.99	1.589 ± 0.039 ^bc^
**Galanthamine**	y = 834.84x + 14.195 R^2^ = 0.99	0.043 ± 0.008 ^d^

The inhibitory effect of standard solution of (+)-catechin (**C**), (-)-epicatechin (**EC**), (-)-epigallocatechin (**EGC**), (-)-epicatechin-3-gallate (**ECG**), and (-)-epigallocatechin-3-gallate (**EGCG**) in comparison to galanthamine was determined within the concentration range of 1.0–0.01 mM. Data are expressed as means ± standard deviation (n = 6). Equation of the linear regression was used for IC_50_ calculation. Different superscript letters (a–d) within a column indicate significant differences at *p* < 0.05.

**Table 4:** The inhibitory activity of catechins against AGE formation (IC_50_).

Compound	BSA/Glucose Model System		BSA/MGO Model System	
	Equation of the Linear Regression	IC_50_ (mM)	Equation of the Linear Regression	IC_50_ (mM)
**C**	y = 50.27x + 24.917 R^2^ = 0.99	0.499 ± 0.023 ^a^	y = 41.273x + 18.533 R^2^ = 0.94	0.762 ± 0.045 ^a^
**EC**	y = 43.923x + 31.319 R^2^ = 0.94	0.425 ± 0.017 ^b^	y = 65.682x + 14.52 R^2^ = 0.96	0.540 ± 0.038 ^b^
**EGC**	y = 55.286x + 27.611 R^2^ = 0.96	0.405 ± 0.011 ^b^	y = 61.826x + 24.59 R^2^ = 0.96	0.411 ± 0.027 ^c^
**ECG**	y = 43.175x + 34.501 R^2^ = 0.82	0.359 ± 0.016 ^c^	y = 86.862x + 4.888 R^2^ = 0.99	0.523 ± 0.037 ^b^
**EGCG**	y = 49.632x + 33.572 R^2^ = 0.88	0.331 ± 0.036 ^c^	y = 62.904x + 27.851 R^2^ = 0.98	0.352 ± 0.021 ^c^
**AG**	y = 56.077x + 25.52 R^2^ = 0.94	0.436 ± 031 ^ab^	y = 62.003x + 17.164 R^2^ = 0.99	0.529 ± 0.031 ^b^

Data are expressed as means ± standard deviation (n = 6). Equation of the linear regression was used for IC_50_ calculation. Different superscript letters (a–c) within a column related to a respective model system indicate significant differences at *p* < 0.05.

## Data Availability

The original contributions presented in this study are included in the article. Further inquiries can be directed to the corresponding author.

## Supplementary Materials

The following supporting information can be downloaded at: https://www.mdpi.com/article/10.3390/molecules31081328/s1, Figure S1. Equations of the linear regression against AGEs formation.

## References

[1] Musial C., Kuban-Jankowska A., Gorska-Ponikowska M. (2020). Beneficial Properties of Green Tea Catechins. Int. J. Mol. Sci..

[2] Cabrera C., Artacho R., Gimenez R. (2006). Beneficial Effects of Green Tea—A Review. J. Am. Coll. Nutr..

[3] Masek A., Chrzescijanska E., Latos M., Zaborski M., Podsedek A. (2017). Antioxidant and antiradical properties of green tea extract compounds. Int. J. Electrochem. Sci..

[4] Tadano N., Du C., Yumoto F., Morimoto S., Ohta M., Xie M., Nagata K., Zhan D., Lu Q., Miwa Y. (2010). Biological Actions of Green Tea Catechins on Cardiac Troponin C. Br. J. Pharmacol..

[5] Singh B.N., Shankar S., Srivastava R.K. (2011). Green Tea Catechin, Epigallocatechin-3-Gallate (EGCg), Mechanisms, Perspectives and Clinical Applications. Biochem. Pharmacol..

[6] Gupta D.A., Bhaskar D.J., Gupta R.K. (2014). Green tea: A review on its natural anti-oxidant therapy and cariostatic benefits. Biol. Sci. Pharm. Res..

[7] Schulze J., Melzer L., Smith L., Teschke R. (2017). Green Tea and Its Extracts in Cancer Prevention and Treatment. Beverages.

[8] Yang Y., Zhang T. (2019). Antimicrobial Activities of Tea Polyphenol on Phytopathogens: A Review. Molecules.

[9] Beltz L.A., Bayer D.K., Moss A.L., Simet I.M. (2006). Mechanisms of cancer prevention by green and black tea polyphenols. Anticancer Agents Med. Chem..

[10] Kuban-Jankowska A., Kostrzewa T., Lo-Bosco G., Lo-Celso F., Musial C., Barone G., Gorska-Ponikowska M. (2020). Green Tea Catechins Induce Inhibition of PTP1B Phosphatase in Breast Cancer Cells with Potent Anti-Cancer Properties: In Vitro Assay, Molecular Docking, and Dynamics Studies. Antioxidants.

[11] Yang C.S., Lambert J.D., Sang S. (2009). Antioxidative and anticarcinogenic activities of tea polyphenols. Arch. Toxicol..

[12] Subramani C., Natesh R.K. (2003). Molecular mechanisms and biological implications of green tea polyphenol, (-)-epigallocatechin-3-gallate. Int. J. Pharma Biosci. Technol..

[13] Botten D., Fugallo G., Fraternali F., Molteni C. (2015). Structural Properties of Green Tea Catechins. J. Phys. Chem. B.

[14] Ambigaipalan P., Young W., Shahidi F. (2020). Epigallocatechin (EGC) esters as potential sources of antioxidants. Food Chem..

[15] Baranowska M., Suliborska K., Chrzanowski W., Kusznierewicz B., Namieśnik J., Bartoszek A. (2018). The relationship between standard reduction potentials of catechins and biological activities involved in redox control. Redox Biol..

[16] Zielińska D., Starowicz M., Wronkowska M., Zieliński H. (2025). Multifaceted Biological Activity of Rutin, Quercetin, and Quercetin’s Glucosides. Molecules.

[17] Zielińska D., Starowicz M., Wronkowska M., Zieliński H. (2025). Angiotensin-Converting Enzyme Inhibitory Activity of Selected Phenolic Acids, Flavonoids, Their O-Glucosides, and Low-Molecular_Weight Phenolic Metabolites in Relation to Their Oxidation Potentials. Metabolites.

[18] Persson I.-A.-L., Josefsson M., Persson K., Andersson R.G.G. (2006). Tea flavanols inhibit angiotensin-converting enzyme activity and increase nitric oxide production in human endothelial cells. J. Pharm. Pharmacol..

[19] Shukor N.A., Camp J.V., Gonzales G.B., Staljanssens D., Struijs K., Zotti M.J., Raes K., Smagghe G. (2013). Angiotensin-Converting Enzyme Inhibitory Effects by Plant Phenolic Compounds: A Study of Structure Activity Relationships. J. Agric. Food Chem..

[20] Magalhaes L.M., Segundo M.A., Reis S., Lima J.L.F.C. (2008). Methodological aspects about in vitro evaluation of antioxidant properties. Anal. Chim. Acta.

[21] Zielińska D., Zieliński H. (2011). Antioxidant activity of flavone C-glucosides determined by updated analytical strategies. Food Chem..

[22] Rebelo M.J., Rego R., Ferreira M., Oliveira M.C. (2013). Comparative study of the antioxidant capacity and polyphenol content of Douro wines by chemical and electrochemical methods. Food Chem..

[23] Vilas-Boasa A., Valderrama P., Fontesc N., Geraldoa D., Bento F. (2019). Evaluation of total polyphenol content of wines by means of voltammetric techniques: Cyclic voltammetry vs differential pulse voltammetry. Food Chem..

[24] Chiorcea-Paquim A.M., Enache T.A., De Souza Gil E., Oliveira-Brett A.M. (2020). Natural phenolic antioxidants electrochemistry: Towards a new food science methodology. Compr. Rev. Food Sci. Food Saf..

[25] Sochor J., Dobes J., Krystofova O., Ruttkay-Nedecky B., Babula P., Pohanka M., Jurikova T., Zitka O., Adam V., Klejdus B. (2013). Electrochemistry as a Tool for Studying Antioxidant Properties. Int. J. Electroch. Sci..

[26] Gupta R., Guptha S. (2010). Strategies for initial management of hypertension. Indian J. Med. Res..

[27] Lin K., Zhang L., Han X., Meng Z., Zhang J., Wu Y., Cheng D. (2018). Quantitative structure-activity relationship modeling coupled with molecular docking analysis in screening of angiotensin I-converting enzyme inhibitory peptides from qula casein hydrolysates obtained by two-enzyme combination hydrolysis. J. Agric. Food Chem..

[28] Fujita H., Yokoyama K., Yoshikawa M. (2000). Classification and antihypertensive activity of angiotensin I-converting enzyme inhibitory peptides derived from food proteins. J. Food Sci..

[29] Kim S.K., Byun H.G., Park P.J., Shahidi F. (2001). Angiotensin I converting enzyme inhibitory peptides purified from bovine skin gelatin hydrolysate. J. Agric. Food Chem..

[30] Mattson M.P. (2004). Pathways towards and away from Alzheimer’s disease. Nature.

[31] Houghton P.J., Howes M.J. (2005). Natural products and derivatives affecting neurotransmission relevant to Alzheimer’s and Parkinson’s disease. Neurosignals.

[32] Conforti L., Adalbert R., Coleman M.P. (2007). Neuronal death: Where does the end begin?. Trends Neurosci..

[33] Okello E.J., Leylabi R., McDougall G.J. (2012). Inhibition of acetylcholinesterase by green and white tea and their simulated intestinal metabolites. Food Funct..

[34] Wimo A., Winblad B., Aguero-Torres H., von Strauss E. (2003). The magnitude of dementia occurrence in the world. Alzheimer Dis. Assoc. Disord..

[35] Uriate-Pueyo I., Calvo M.I. (2011). Flavonoids as Acetylocholinesterase Inhibitors. Curr. Med. Chem..

[36] Adewusi E.A., Steenkamp V. (2011). In vitro screening for acetylcholinesterase inhibition and antioxidant activity of medicinal plants from southern Africa. Asian Pac. J. Trop. Med..

[37] Williams P., Sorribas A., Howes M.J.R. (2011). Natural products as a source of Alzheimer’s drug leads. Nat. Prod. Rep..

[38] Oei S., Millar C.L., Lily T.N.N., Mukamal K.J., Kiel D.P., Lipsitz L.A., Hannan M.T., Sahni S. (2023). Higher intake of dietary flavonols, specifically dietary quercetin, is associated with lower odds of frailty onset over 12 years of follow-up among adults in the Framingham Heart Study. Am. J. Clin. Nutr..

[39] Barbosa Filho J.M., Medeiros K.C.P., Diniz M.F.F.M., Batista L.M., Athayde-Filho P.F., Dilva M.S., Cunha E.V.L., Almeida I.R.G.S., Quintans-Junior L.J. (2006). Natural products inhibitors of the enzyme acetylcholinesterase. Res. Bras. Farmacogn..

[40] Ahmed N. (2005). Advanced glycation endproducts—Role in pathology of diabetic complications. Diabetes Res. Clin. Pract..

[41] Buetler T. (2007). The Health Risks of Dietary Advanced Glycation End-products. Mol. Nutr. Food Res..

[42] Zhang Q., Wang Y., Fu L. (2020). Dietary advanced glycation end-products: Perspectives linking food processing with health implications. Compr. Rev. Food Sci. Food Saf..

[43] Lunceford N., Gugliucci A. (2005). Ilex paraguariensis extracts inhibit AGE formation more efficiently than green tea. Fitoterapia.

[44] Pashikanti S., de Alba D.R., Boissonneault G.A., Cervantes-Laurean D. (2010). Rutin metabolites: Novel inhibitors of nonoxidative advanced glycation end products. Free Radic. Biol. Med..

[45] Choi J.S., Islam M.N., Ali M.Y., Kim E.J., Kim Y.M., Jung H.A. (2014). Effects of C-glycosylation on anti-diabetic, anti-Alzheimer’s disease and anti-inflammatory potential of apigenin. Food Chem. Toxicol..

[46] Grzesik M., Naparło K., Bartosz G., Sadowska-Bartosz I. (2018). Antioxidant properties of catechins: Comparison with other antioxidants. Food Chem..

[47] Chevion S., Roberts M.A., Chevion M. (2000). The use of cyclic voltammetry for the evaluation of antioxidant capacity. Free Radic. Biol. Med..

[48] Wang W., Le T., Wang W.-W., Yin J.-F., Jiang H.-Y. (2023). The Effects of Structure and Oxidative Polymerization on Antioxidant Activity of Catechins and Polymers. Foods.

[49] Blasco A.J., Rogerio M.C., Gonzalez M.C., Escarpa A. (2005). “Electrochemical Index” as a screening method to determine “total polyphenolics” in foods: A proposal. Anal. Chim. Acta.

[50] Yang B., Kotani A., Arai K., Kusu F. (2001). Relationship of Electrochemical Oxidation of Catechins on Their Antioxidant Activity in Microsomal Lipid Peroxidation. Chem. Pharm. Bull..

[51] Masek A., Chrzescijanska E., Zaborski M. (2015). Electrochemical Properties of Catechin in Non-Aqueous Media. Int. J. Electrochem. Sci..

[52] Lopez-Sendon J., Swedberg K., McMurray J., Tamargo J., Maggioni A.P., Dargie H., Tendera M., Waagstein F., Kjekshus J., Lechat P. (2004). Expert consensus document on angiotensin converting enzyme inhibitors in cardiovascular disease. The Task Force on ACE-inhibitors of the European Society of Cardiology. Eur. Heart J..

[53] Ensafi A.A., Rezaei B., Zare Z.M., Maleh K. (2011). Highly Selective and Sensitive Voltammetric Sensor for Captopril Determination Based on Modified Multiwall Carbon Nanotubes Paste Electrode. J. Braz. Chem. Soc..

[54] Acharya J., Karak S., De B. (2016). Metabolite profile and bioactivity of *Musa paradisiaca* L. flower extracts. J. Food Biochem..

[55] Balasuriya B.W.N., Rupasinghe H.P.V. (2011). Plant flavonoids as angiotensin converting enzyme inhibitors in regulation of hypertension. Funct. Food Health Dis..

[56] Guerrero L., Castillo J., Quinones M., Garcia-Vallve S., Arola L., Pujadas G., Muguerza B. (2012). Inhibition of angiotensin-converting enzyme activity by flavonoids: Structure-activity relationship studies. PLoS ONE.

[57] Paiva L., Lima E., Marcone M., Baptista J. (2023). Angiotensin I-converting enzyme (ACE) inhibition and biological activities of green and black tea samples from Azorean *Camellia sinensis*. J. Funct. Foods.

[58] Bores G.M., Huger F.P., Petko W., Mutlib A.E., Camacho F., Rush D.K., Selk D., Wolf V., Kosley R.W., Davis L. (1996). Pharmacological evaluation of novel Alzheimer’s disease therapeutics: Acetylcholinesterase inhibitors related to galanthamine. J. Pharmacol. Exp. Ther..

[59] Heinrich M., Teoh H.L. (2004). Galanthamine from snowdrop—The development of a modern drug against Alzheimer’s disease from local Caucasian knowledge. J. Ethnopharmacol..

[60] Zielińska D., Zieliński H. (2025). Multifaceted Biological Activity of Selected Flavone C-Monoglucosides. Int. J. Mol. Sci..

[61] Şenol F.S., Orhan İ., Yilmaz G., Çiçek M., Şener B. (2010). Acetylcholinesterase, butyrylcholinesterase, and tyrosinase inhibition studies and antioxidant activities of 33 *Scutellaria* L. taxa from Turkey. Food Chem. Toxicol..

[62] Sakakibara H., Honda Y., Nakagawa S., Ashida H., Kanazawa K. (2003). Simultaneous determination of all polyphenols in vegetables, fruits, and teas. J. Agric. Food Chem..

[63] Tian J., Geiss C., Zarse K., Madreiter-Sokolowski C.T., Ristow M. (2021). Green tea catechins EGCG and ECG enhance the fitness and lifespan of *Caenorhabditis elegans* by complex I inhibition. Aging.

[64] Okello E.J., Mather J. (2020). Comparative Kinetics of Acetyl- and Butyryl-Cholinesterase Inhibition by Green Tea Catechins/Relevance to the Symtomic Treatment of Alzheimer’s Disease. Nutrients.

[65] Mrabti H.N., Jaradat N., Fichtali I., Ouedrhiri W., Jodeh S., Ayesh S., Cherrah Y., Faouzi M.E.A. (2018). Separation, Identification, and Antidiabetic Activity of Catechin Isolated from *Arbutus unedo* L. Plant Roots. Plants.

[66] Sentandreu M.Á., Toldrá F. (2006). A rapid, simple and sensitive fluorescence method for the assay of angiotensin-I converting enzyme. Food Chem..

[67] Eldeen I.M.S., Elgorashi E.E., Staden J. (2005). Antibacterial, anti-inflammatory, anti-cholinesterase and mutagenic effects of extracts obtained from some trees used in South African traditional medicine. J. Ethnopharmacol..

[68] Lo C.Y., Li S., Tan D., Pan M.H., Sang S., Ho C.T. (2006). Trapping reactions of reactive carbonyl species with tea polyphenols in simulated physiological conditions. Mol. Nutr. Food Res..

[69] Giménez-Bastida J.A., Zieliński H., Piskula M., Zielińska D., Szawara-Nowak D. (2017). Buckwheat bioactive compounds, their derived phenolic metabolites and their health benefits. Mol. Nutr. Food Res..

